# Leprosy reactions: Unraveling immunological mechanisms underlying tissue damage in leprosy patients

**DOI:** 10.1093/femspd/ftae013

**Published:** 2024-05-28

**Authors:** Héctor Serrano-Coll, Eric L Wan, Lina Restrepo-Rivera, Nora Cardona-Castro

**Affiliations:** Instituto Colombiano de Medicina Tropical-Universidad CES, Medellín 055450, Colombia; Georgetown University School of Medicine, 3900 Reservoir Rd NW, Washington DC 200072, United States; Instituto Colombiano de Medicina Tropical-Universidad CES, Medellín 055450, Colombia; Instituto Colombiano de Medicina Tropical-Universidad CES, Medellín 055450, Colombia

**Keywords:** immunology, mycobacterium infections, hansen's disease, disability, hypersensitivity

## Abstract

Leprosy is a chronic granulomatous infectious and disabling disease caused by two mycobacteria, Mycobacterium leprae and Mycobacterium lepromatosis. Acute inflammatory responses, known as leprosy reactions, are significant contributors to disabilities. Three types of leprosy reactions have been identified based on excessive cytokine release (e.g. type 1) or the accumulation of immune complexes in tissues inducing multiorgan damage (e.g. types 2 and 3). The type of leprosy reaction has implications on treatment and management strategies, yet are not well understood by health workers caring for leprosy patients. We attempt to describe the immunologic mechanisms behind the different leprosy reactions and the rationale for tailoring clinical treatment and management to the particular type of leprosy reaction based on the underlying immunologic situation.

## Teaser

Leprosy, a disabling disease, triggers inflammatory reactions affecting patients. Understanding these reactions is crucial for better treatment, but it's not well-known among healthcare workers caring for leprosy patients.

## List of abbreviations

LRs: Leprosy reactions.
MDT: Multidrug therapy.
T1R: Type 1 leprosy reactions.
T2R: Type 2 leprosy reactions.
T3R: Type 3 leprosy reactions.
RR: Reversal reactions.
TL: T lymphocytes.
WHO: World Health Organization.
Tregs: T regulatory cells.
ERN: Erythema nodosum leprosum.
SC: Schwann cell.
NEN: Necrotizing erythema nodosum.
MDT-MB: Multibacillary-Multidrug therapy.

## Introduction

Leprosy is a chronic granulomatous infectious disease caused by two types of mycobacteria: *Mycobacterium leprae* and *Mycobacterium lepromatosis* (Cardona-Castro et al. 2022). Approximately 5% of the world's population is susceptible to infection, which, along with the immunological response to infection, can damage the eyes (e.g. lagophthalmos, keratitis, iritis, acute and chronic uveitis, secondary glaucoma), cause neuropathy with functional and sensory loss, and produce deformities and ulcers to the hands and feet (Rathod et al. 2020, Deschênes 2023). Additionally, the infection and immunologic reaction to the bacterium can cause canonical neurological and dermatologic symptoms (e.g. erythematous macules, panniculitis, inflamed plaques, neuritis) (Nunzi et al. 2020).

On the other hand, leprosy presents a wide clinical spectrum. Indeterminate leprosy, the initial form of the disease, represents a stage where symptoms are subtle and not clearly defined. Tuberculoid leprosy is characterized by localized skin lesions and nerve damage, while lepromatous leprosy is typified by widespread skin nodules and profound nerve involvement. Borderline leprosy, positioned between these two extremes (tuberculoid and lepromatous), exhibits a combination of symptoms, featuring varying degrees of skin lesions and nerve damage (Nunzi et al. 2020).

Current treatment to rid the patient of the bacterial infection involves multidrug therapy (MDT) comprising dapsone, clofazimine, and rifampicin for six doses (paucibacillary forms) for PB and 12 doses (multibacillary forms) (Nunzi et al. 2020). While the MDT resolves the bacterial infection in most cases, it is a common misunderstanding that patients after MDT do not have further immunological concerns and thus need not be followed clinically. The destruction of the bacterium by the MDT is not clean but instead leaves behind remnants of bacterial cells. These particles trigger a series of acute inflammatory episodes known as leprosy reactions (LRs), causing further short-term and long-term damage to patients, aggravating dermatologic, neurologic, and indeed multi-system symptoms discussed further below. Therefore, it is important to consider leprosy as more than an infectious disease; the long-term sequelae are often related to LRs from the perspective of an immunological disorder.

Importantly, LRs are heterogeneous in pathophysiology and clinical presentation and thus require different approaches to clinical treatment and management (Wu and Boggild 2016). LRs cause variable dermatologic damage and pain, limiting the activities of patients, and also can be unpredictable both in frequency, onset, and response to therapy. They can also exacerbate the stigma and socio-economic losses that come with a leprosy diagnosis (Putri et al. 2022). Thus, it is important that treatment and response monitoring account for the underlying pathophysiology as well as the stigma and socioeconomic burden caused by LRs. Following the patient, often at more frequent intervals, is critical to ensure medical adherence and the prevention of relapse.

## General overview of leprosy reactions

LRs are commonly considered immune hypersensitivity responses that can manifest at any time before, during, or after completing leprosy multidrug therapy (MDT) (Pitta et al. 2022, Putri et al. 2022). To date, three distinct types of LR have been described: Type 1 leprosy reactions (T1R) trigger inflammation in the skin and peripheral nerves due to an excessive release of cytokines due to an increased Th1 and Th17 cellular immune response (i.e. type IV hypersensitivity) (Mitra 2018). Type 2 leprosy reactions (T2R) and type 3 leprosy reactions (T3R) are associated with multi-organ and multi-tissue inflammatory responses, with T2R could involve type III hypersensitivity (i.e. antigen-antibody immune complex deposition) or an increase in Th17 cells, along with a decrease in T regulatory cells (Tregs) (Luo et al. 2021); T3R could be associated with type III hypersensitivity (Velarde-Félix et al. 2016). These systemic clinical manifestations are associated with the formation and accumulation of immune complexes within affected tissues, leading to disabilities and impacting the quality of life of the leprosy patients (Fonseca AB de et al. 2017).

For this reason, LRs are a critical issue in the context of leprosy, and we propose that understanding their immunological and cellular mechanisms will be crucial in the future to generate biomarkers that facilitate detection and promote the development of new strategies for managing these immune events. This review analyzes and describes the main immunological mechanisms associated with tissue damage induced by LRs.

## Immune response in type 1 leprosy reactions (T1R)

T1R is characterized by an intensified host cellular immune response against either *M. leprae* or *M. lepromatosis* (Antunes et al. 2019). The heightened immune response not only leads to the elimination of the mycobacteria but also results in collateral damage to the infected tissues, notably the skin and peripheral nerves (Nery JA da et al. 2013). T1R can occur in approximately one-third of patients with borderline leprosy and is often called a “reversal reaction” due to the hyperergic immune response mimicking a shift in the clinical-histological Ridley-Jopling classification as patients move from a borderline spectrum to a tuberculoid leprosy form (Naafs and van Hees 2016, Froes et al. 2022).

**Figure 1.**
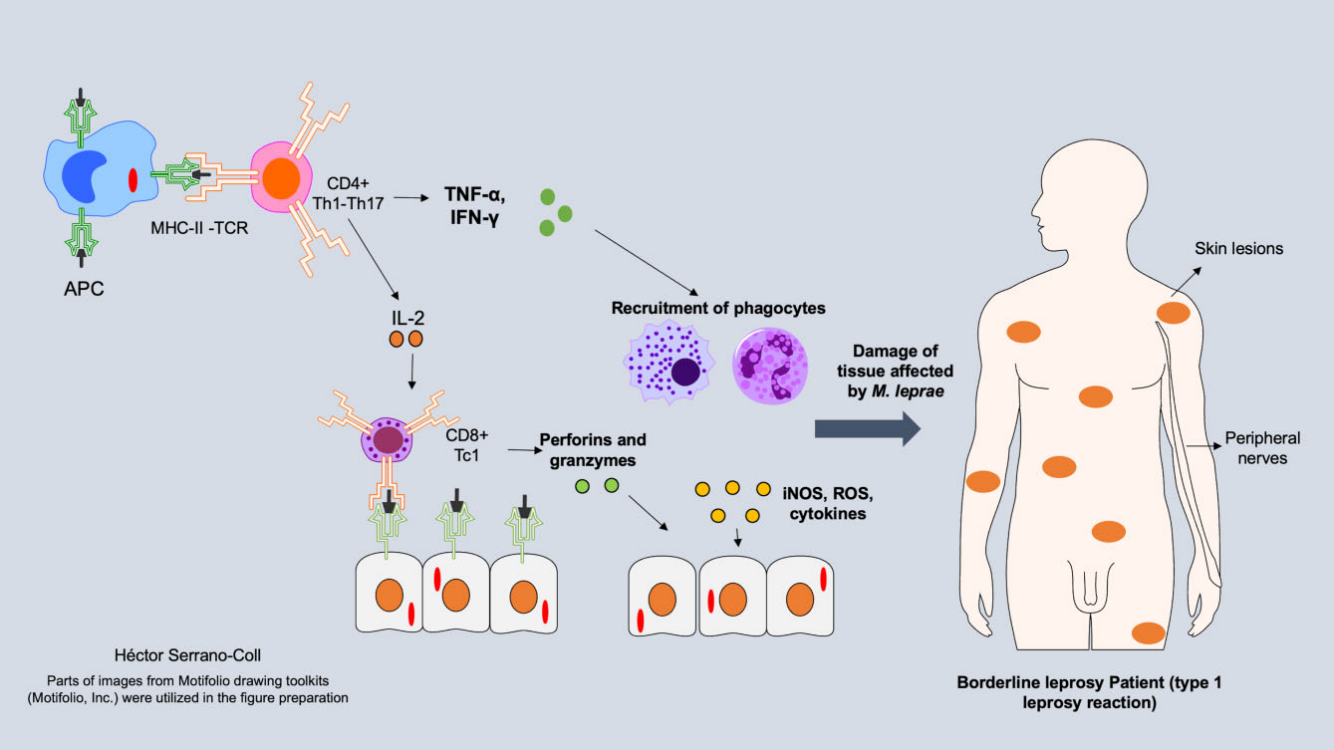
Immune mechanism of tissue damage during T1R. This figure shows an increase in the activity of CD4 T cells with Th1 and Th17 patterns and CD8 Tc1 cells, which induce a proinflammatory environment, resulting in collateral damage to tissues affected by *M. leprae* (skin and peripheral nerves).

RR is a Type IV hypersensitivity reaction that induces a heightened activation of T lymphocytes (TL) featuring a Th1 and Th17 effector pattern (Serrano-Coll et al. 2018). Constistent with Th1 activity, TLs stimulate an increased release of IL-2, which creates a robust clonal expansion of CD8 TLs following a cytotoxic Tc1 pattern (Dewi et al. 2023). When CD8 TLs encounter residual mycobacterial antigens presented on major histocompatibility complex class I (MHC-I) molecules within infected cells in the skin and peripheral nerves, they initiate the release of perforins and granzymes, resulting in neuritis and panniculitis in the subcutaneous adipose tissue of the skin (de Oliveira et al. 2013, Abbas et al. 2015). Moreover, this inflammatory process in the skin and peripheral nerves is further intensified by Th1 and Th17 CD4 TLs secreting IL-17, TNF-α, and IFN-γ (Nery JA da et al. 2013, Naafs and van Hees 2016, Fonseca AB de et al. 2017). These cytokines recruit neutrophils and macrophages, which adopt the classical M1 pro-inflammatory phenotype, thereby exacerbating tissue damage (Schmitz et al. 2019). In Fig. 1, we describe canonical tissue damage mediated by T1R immunological mechanisms.

Interestingly, the T-cell dependency of Type 1 reactions created a concern that Type 1 reactions could be triggered by vaccinating leprosy patients against SARS-CoV-2 with the BNT162b2 and CoronaVac vaccines. There have been at least 7 cases of leprosy patients receiving SARS-CoV-2 vaccines and then presenting with a Type 1 reaction. However, in a UK retrospective cohort study, de Barros et al. (de Barros et al. 2023) could not to establish a relation between SARS-CoV-2 vaccination and an increase in the incidence of RR in leprosy patients or in tuberculoid leprosy cases in individuals with latent infections. However, further studies are needed to explore the association between SARS-CoV-2 vaccination and the development of LRs.

Finally, it is crucial to note that the heightened cellular immune response in T1R on peripheral nerves can quickly induce deformities and disabilities in the eyes, hands, and feet (Serrano-Coll et al. 2018), affecting the function and quality of life of these patients. The frequency of nerve damage during T1Rs can very high, ranging from between 43% and 73%, and underlines the importance of monitoring and treatment to prevent lifelong disability (Naafs and van Hees 2016). Nerve damage can also occur during T2R and can even appear alone in the context of no ongoing reaction.

## Immune response in type 2 leprosy reactions (T2R) or erythema nodosum leprosum

T2R is an immune event in leprosy patients that can be explained through various immune mechanisms, including 1. Deposition of immune complexes at the tissue level, 2. Immune switch from a Th2 to a Th1 and Th17 response (Fonseca AB de et al. 2017).

In contrast to the hyperergic immunopathogenesis of T1R, T2R is relatively anergic despite being mediated by immune complexes (type III hypersensitivity) (Mitra 2017). Not surprisingly, T2R more commonly occurs in the anergic spectrums of leprosy (i.e. lepromatous and borderline lepromatous types of leprosy). These clinical types are associated with high bacillary indices ranging from 4 + to 6 + on the logarithmic Ridley scale out of a maximum of 6 +. Patients with a high bacterial load of *M. leprae* or *M. lepromatosis* are more likely to have T2R and not T1R (Nunzi et al. 2020).

Moreover, these changes in the host immune response can be induced by infections, pregnancy, and other factors associated with the host, which could induce a humoral immune response and the further release of antibodies that may interact with mycobacterial antigens resulting in immune complexes (Galeano et al. 2023). Our hypothesis is that these immune complexes could recruit basophils, which would degranulate and release vasoactive amines promoting vasodilation and increasing vascular endothelial permeability, thus facilitating the deposition of immune complexes in different tissues.

**Figure 2.**
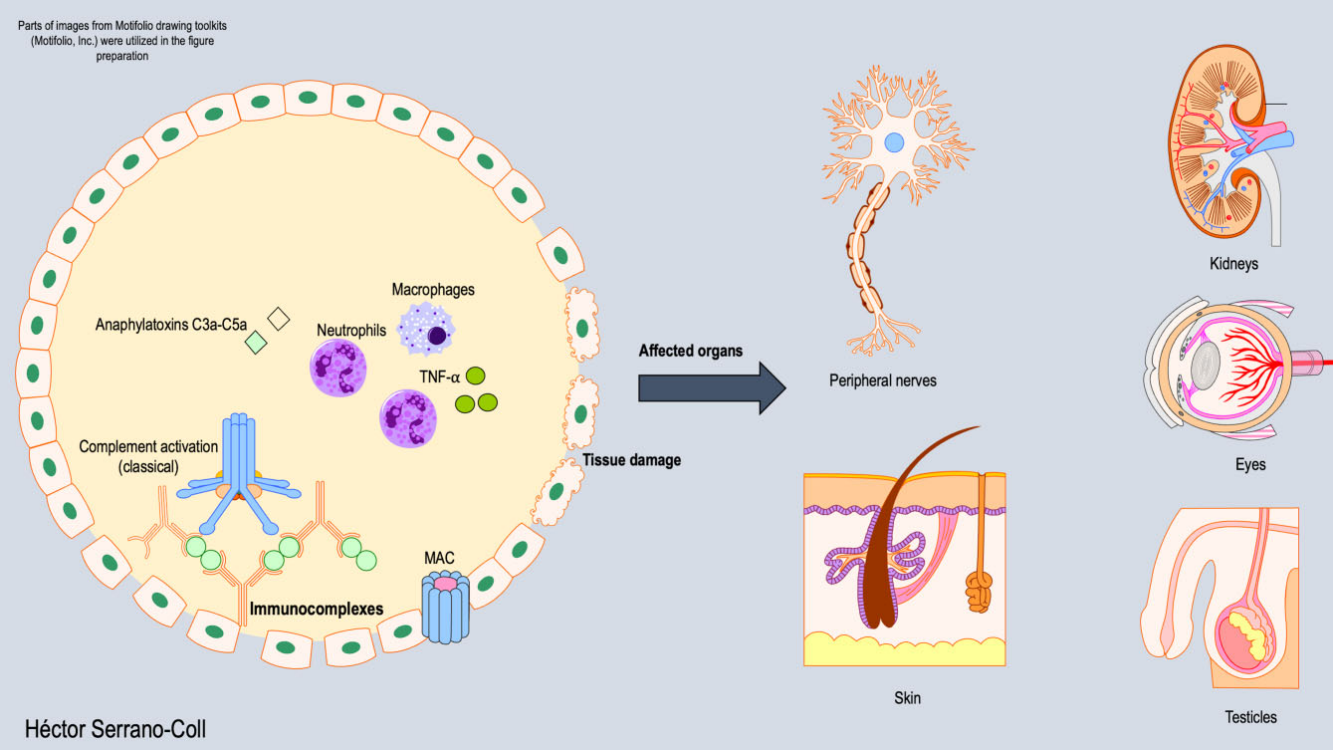
Immune mechanism of tissue damage during T2R. This figure shows that the formation of immune complexes activates the classical complement pathway, leading to the recruitment of phagocytes that induce damage in various tissues (nerves, skin, kidneys, eyes, and testicles).

Once immune complexes have been deposited within the tissue, they can activate the classical complement pathway and trigger tissue damage through two additional mechanisms (Dupnik et al. 2015). The first mechanism is direct, as activation of the complement pathway initiates a proteolytic cascade that promotes the formation of the membrane attack complex (MAC), resulting in tissue damage (Bahia El Idrissi et al. 2016, Bahia El Idrissi et al. 2017). The second mechanism is indirect and mediated by anaphylatoxins (C3a and C5a) released during complement activation, which induces the recruitment of neutrophils and macrophages. These phagocytes recognize immune complexes and opsonin C3b deposited in the tissues, resulting in production of TNF-α and reactive oxygen species (ROS) and causing tissue damage that can clinically manifest as panniculitis, peripheral neuropathies, epididymal-orchitis, glomerulonephritis, myositis, arthralgia, and hepatomegaly (Serrano-Coll et al. 2018, Antunes et al. 2022). Figure 2 illustrates the tissue damage mediated by T2R. Besides, another immunological mechanism implicated in T2R is the decrease in the populations of Tregs expressing TGF-β (Gomes de Castro et al. 2022). These alterations in cytokine profiles induce the release of IL-17 and IL-6 by Th17 T cells, contributing to increased tissue damage (Gomes de Castro et al. 2022).

T2R can occur before, during, or after MDT (Singh et al. 2014) and most often occur during treatment (Putinatti MS de et al. 2014) because MDT, while eliminating the live bacterium, increases the number of fragmented bacilli and circulating antigens (Antunes et al. 2016), thereby facilitating the formation of immune complexes. Furthermore, MDT has been linked to a shift from Th2 to Th1 and Th17 responses, potentially increasing the likelihood of T2R development in the host (Fonseca AB de et al. 2017). While MDT may trigger these reactions, adhering to the treatment regimen outlined by the WHO guidelines remains crucial to ensuring complete elimination of the bacterium in the patient.

## Immunopathogenesis of type 3 leprosy reaction (T3R)

T3R, or Lucio's phenomenon, frequently occurs within the Th2 or anergic spectrum of leprosy, particularly in cases associated with diffuse lepromatous leprosy (DLL) (i.e. “pretty leprosy” or Lucio's leprosy) and nodular lepromatous leprosy (Jurado et al. 2015, Ya et al. 2021). Furthermore, this reaction is associated with rapid, severe clinical evolution and high mortality (Frade et al. 2022). T3R or Lucio's phenomenon could be regarded as a variant of T2R known as necrotizing erythema nodosum (NEN) (Ranugha et al. 2013, Pinheiro et al. 2022).

The T3R could represent a type III hypersensitivity event because patients with DLL have a high antigenic load (6+) (Cruz et al. 2023). The elevated antigenic burden may precipitate the generation of immune complexes, thus promoting activation of the classical complement pathway, as previously noted. However, this hypothesis requires further validation (Jurado et al. 2015, Sharma et al. 2019). The accumulation of immune complexes in medium and small-sized vessels (Cruz et al. 2023) triggers phagocyte migration, resulting in high secretion levels of proinflammatory cytokines (TNF-α and IFN-γ) (Misra et al. 2014, Polycarpou et al. 2017). Additionally, thrombus formation due to platelet aggregation in medium and small-sized vessels induces a vasculonecrotic reaction, clinically presenting as multiple, potentially large and extensive skin ulcers (Tajalli and Wambier 2021).

**Figure 3.**
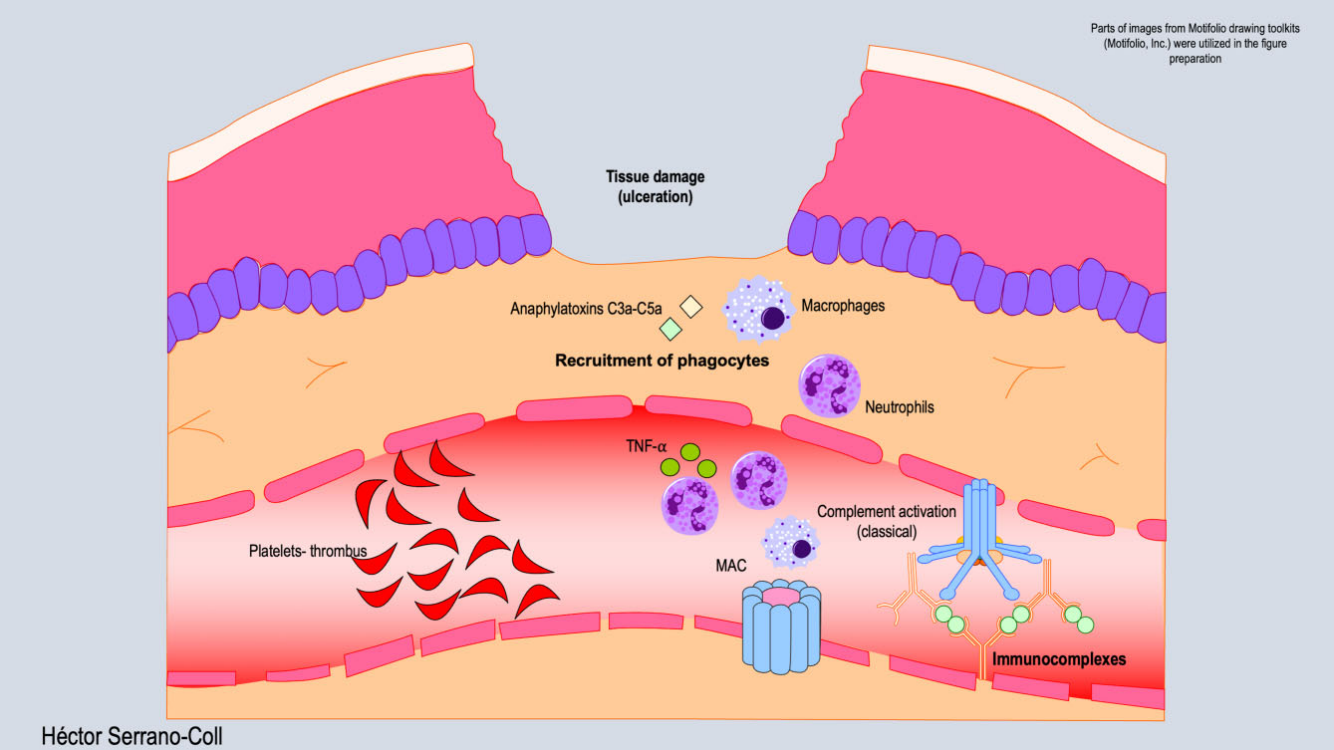
Immune mechanism of ulceration during T3R. This figure shows that the formation of immune complexes can activate the classical complement pathway, leading to the recruitment of predominantly polymorphonuclear phagocytes, along with significant platelet aggregation in blood vessels, forming thrombus and inducing necrotic vasculopathy.

Accumulation of immune complexes in the medium and small-sized vessels (Misra et al. 2014) leads to phagocyte migration, therefore high secretion levels of proinflammatory cytokines (TNF-α e IFN-γ) than the T2R in a classic erythema nodosum leprosum (ENL). Thrombus formation due to platelet aggregation in medium and small-size vessels induces a vasculonecrotic reaction that would manifest clinically as multiple skin ulcers, which can be quite large and extensive (Sharma et al. 2019). Figure 3 describes T3R-mediated skin tissue damage.

## Clinical manifestations of leprosy reactions and their immunologic explanation

**Figure 4:**
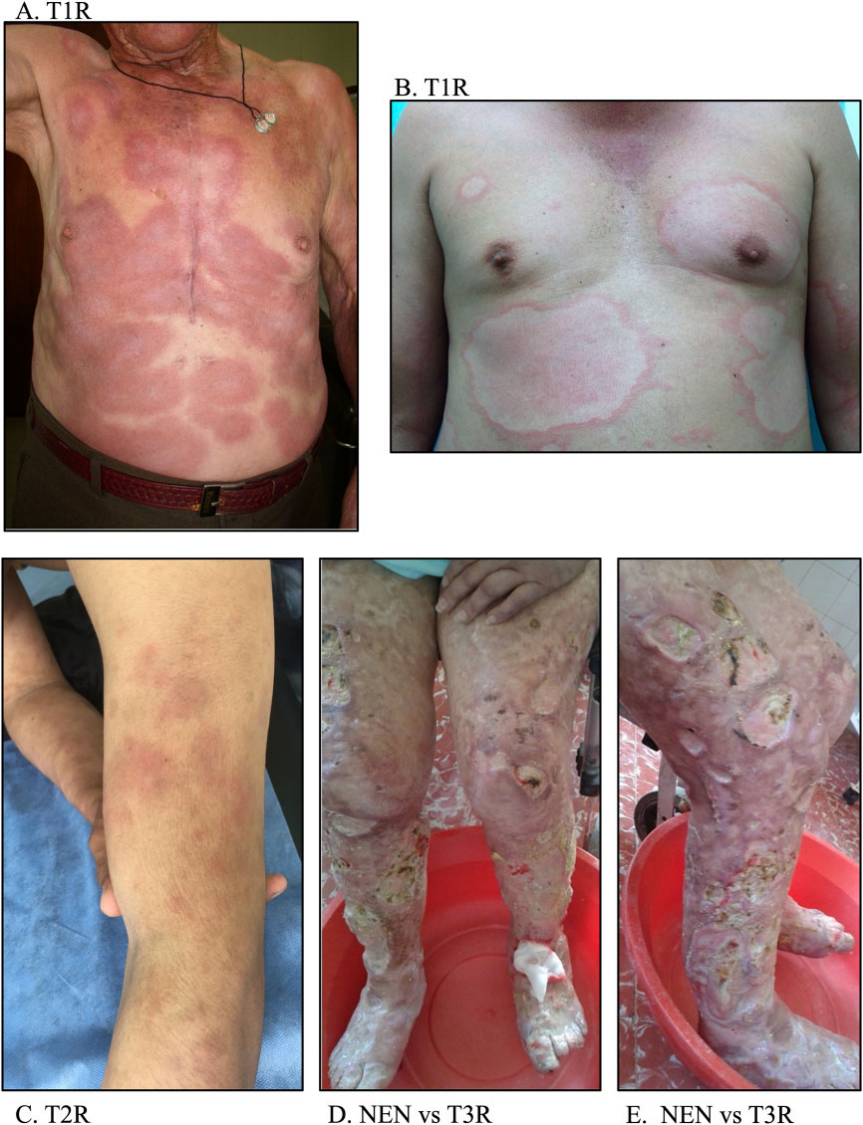
A-E. Skin manifestations of leprosy reactions. A-B. These images show inflammation and erythema in the skin lesions. C. This image shows subcutaneous nodules in a patient with T2R. D-E. These figures show ulcerative lesions in lower limbs (NEN vs T3R). NEN: Necrotizing erythema nodosum, T3R: Type 3 leprosy reactions.

The T1R or RR is an acute inflammatory episode causing sudden worsening of existing skin lesions and nerve pain due to heightened immune response (Stefani et al. 2009). Besides, these pathological events occur as indirect consequences of these immunological events (Serrano-Coll et al. 2018). The pro-inflammatory environment, fueled by TNF-α and IFN-γ, in conjunction with the LT CD8 Tc1 cytotoxic response, triggers cutaneous inflammation, Schwann cell (SC) damage, and demyelination (Serrano-Coll et al. 2018) (Table 1). The impairment of SCs disrupts the expected propagation of the electrical signal, typically conducted through the nodes of Ranvier via action potentials, leading to compromised depolarization and a subsequent impact on axonal conduction velocity (Alizadeh et al. 2015). Therefore, the demyelinated axons within a SC result in depolarization and a reduction in axonal conduction velocity (Alizadeh et al. 2015). Therefore, the severity of T1R could lead to accelerated demyelination of nerves, potentially resulting in an accelerated progression to grade 2 disability in leprosy patients, characterized by impairments in the eyes, hands, and feet (Scollard 2008, Serrano-Coll et al. 2018). Figure 4 A-B, these figures show skin inflammation in T1R.

**Table 1.** Immune mechanism and treatments for leprosy reactions.

Overall clinical manifestation	Immune mechanism	Clinical Management	Treatment implications in the immune system	References
**Type 1 leprosy reactions**				
Skin inflammation and neuritis	• Increased of the activity of LT CD4 Th1and Th17. • Increased of cytotoxic of LT CD8	Prednisolone (1 mg/Kg/day)	• Reduces the Th1 and Th17 activity of CD4 TL. • Increases the activity of regulatory T cells.	(Nery JA da et al. 2013, Serrano-Coll et al. 2018, Nunzi et al. 2020)
**Type 2 leprosy reactions**				
Panniculitis, multiorgan damage	• Immunocomplex deposit in capillaries. • Classical activation of the complement. • Phagocytes recruitment. • Release of TNF-α.	Thalidomide. Mild forms: 100-200 mg per day. severe forms 300-400 mg per day.	• Preventing the recruitment of phagocytes. • Reduces the release of proinflammatory cytokines (TNF-α, IFN-γ)	(Talhari et al. 2006, Mitra 2017, Upputuri et al. 2020, Vashisht et al. 2022)
**Type 3 leprosy reactions**				
Skin ulceration, multiorgan damage	• Immunocomplex deposit in capillaries. • Classical activation of the complement. • Phagocytes recruitment. • Release of TNF-α. • Blood vessel obstruction.	MB-MDT	• Reduces mycobacterial replication. • Avoid the formation of immune complexes.	(Rocha et al. 2016, Polycarpou et al. 2017, Cruz et al. 2023)

LT: T lymphocytes, MB-MDT: Multibacillary-Multidrug therapy.

The primary manifestations in T2R encompass panniculitis and multi-organ dysfunction (Hafsi and Badri 2023). Panniculitis is characterized by subcutaneous adipose tissue inflammation (panniculus adiposus), presenting clinically as subcutaneous nodules symmetrically distributed on the face, back, and upper limbs (Vashisht et al. 2022). Negera et al. (2017) demonstrated that neutrophilic infiltration was observed in 58.8% of the samples with T2R, along with the presence of eosinophils, mast cells, T cells, and foamy histiocytes throughout the dermis and subcutis, explains the dermal inflammation. (Figure 4C shows nodules in the skin T2R). In cases of T3R or necrotic erythema nodosum, a severe complication of lepromatous leprosy characterized by necrotizing vasculitis, extensive skin lesions and tissue necrosis primarily affect the lower limbs due to phagocyte infiltration and massive deposition of platelets on endothelial and vascular tissues, triggering skin ischemia that can progress to ulcerations and necrosis (Sehgal 2005) (Table 1) and Fig. 4D-E, these figures show the mechanisms of the ulcerative lesions in lower limbs (NEN vs T3R). Nevertheless, a clinically distinguishing feature between these two occurrences is that NEN is often linked to fever and constitutional symptoms, whereas T3R is not (Sharma et al. 2019).

Another pertinent discussion topic is why patients with T2R experience hepatic, kidney, testicular, and ocular injuries when leprosy primarily affects the skin and nerves. To comprehensively address this question, it is essential to recognize that leprosy reactions are associated with an anergic response (Leon et al. 2015). As a result, mycobacterial dissemination in anergic patients affects the skin and nerves and other tissues such as bone marrow, liver, spleen, lymph nodes, lungs, kidneys, eyes, and testes (Gautam et al. 2021). The immune complexes formed through antigen-antibody interactions trigger an inflammatory response and tissue injury (Goulart et al. 2022, Dewi et al. 2023).

## Medical management of leprosy reactions and their immunological explanation

In treating of LRs, prednisolone is critical in controlling T1R (Safa et al. 2009). This medication inhibits the differentiation of CD4 TL into an effector Th1 and Th17 phenotypes. It increases the activity of regulatory T cells, promoting the control of the exacerbated cellular immune response seen in these patients (Della Corte and Morgillo 2019). The immunomodulation induced by this drug results in a reduction of inflammation in the skin and peripheral nerves. The recommended prednisolone dose for managing T1R is 1 mg/kg/day, but in adults, it is advisable to start with a dose ranging from 40-60 mg/day and gradually taper it down (Safa et al. 2009, Nunzi et al. 2020). The duration of this therapy is a subject of debate; however, we believe it should be maintained for one to three months due to the numerous adverse effects associated with long-term use of prednisolone (Nunzi et al. 2020). See Table 1.

Besides, in patients with T1R, it is crucial to consider nerve decompression as an approach to reduce the disability associated with these immunological events, particularly when the nerves demonstrate entrapment within the tunnels of the extremities (Nickerson and Nickerson 2010). Decompression of peripheral nerves leads to a reduction in inflammation, which, from an immunological perspective, would decrease phagocyte chemotaxis and, consequently, the release of proinflammatory cytokines TNF-α, IFN-γ, both by these cells and CD4 and CD8 TL (Schomberg et al. 2012).

On the other hand, Thalidomide has become the drug of choice for treating of T2R. Although this drug was initially synthesized in Germany in the 50 s to address hyperemesis gravidarum, it was withdrawn from the market in the 60 s due to its association with congenital malformations, particularly phocomelia (Talhari et al. 2006). However, in the 21st century, it was described the immunomodulatory properties of this drug and its use in diseases related to type III hypersensitivity reactions, such as T2R (Talhari et al. 2006, Gómez and Castro 2016).

The immunomodulatory effect of thalidomide could be related to the inhibition of TNF-α activity production. The reduction of levels of this cytokine decreases the recruitment of phagocytes, such as macrophages and neutrophils (Upputuri et al. 2020). Therefore, reducing the migration of these cells is related to two beneficial effects in patients with T2R: 1. Preventing the inefficient phagocytosis of immune complexes deposited in host tissues, and 2, inhibiting the release of proinflammatory cytokines (TNF-α, IFN-γ) that are responsible for tissue damage. On the other hand, this medication can reduce the effector activity of CD4 and CD8 TL (Kim et al. 2015). Besides, thalidomide promotes the expression of the transcription factor FoxP3 + in specific subpopulations of CD4 TL, which is crucial in the conversion of these cells into iTreg, which play a vital role in reducing inflammation by suppressing effector patterns such as Th1 and Th17 (Kim et al. 2015). See Table 1.

The dosage of this medication will vary depending on the clinical severity of T2R. In mild cases, defined as ≤ 10 painful nodular lesions with limited systemic manifestations, a recommended dose of thalidomide is 100 to 200 mg daily (Talhari et al. 2006, Upputuri et al. 2020). For erythema nodosum leprosum, Lucio phenomenon, and severe T2R cases characterized by the presence of > 20 painful nodular lesions along with significant systemic manifestations, the thalidomide dosage ranges from 300 to 400 mg per day, with the suggested incorporation of prednisolone at the previously mentioned doses (Talhari et al. 2006, Upputuri et al. 2020). The duration of this medical approach will depend on each patient's clinical progress, and it is always recommended to investigate the presence of an underlying infectious trigger for this immunological event (Galeano et al. 2023). Nevertheless, thalidomide should be avoided in women of childbearing age due to its potential teratogenic effects (Thangaraju et al. 2020). However, it's worth noting that the use of thalidomide in the Lucio phenomenon (T3R) can be a subject of controversy among some authors, as they consider the utility of this medication may be limited and prefer to use Multibacillary-MDT and antibiotics for secondary infections (Rocha et al. 2016). The use of these antibiotics to treat T3R is supported by the fact that the extensive replication of these mycobacteria serves as the trigger for these reactions (Peixoto et al. 2013). Therefore, the administration of these drugs reduces mycobacterial replication and enhances the clinical outcomes in these patients (Peixoto et al. 2013). See Table 1.

## Conclusions and perspective

Leprosy reactions are complex immune-related events that can result in disability or even death. Therefore, enhancing our understanding of leprosy reactions could lead to the discovery of new and valuable biomarkers and immunomodulators for diagnosing and treating of leprosy. In tropical regions, leprosy reactions are often underdiagnosed as they are not typically considered in the differential diagnosis for febrile syndromes, which can hinder medical attention for leprosy patients. Consequently, educating healthcare professionals about these hypersensitivity reactions is crucial since they affect more than half of all leprosy patients.

However, several questions remain unanswered: What are the transcriptomic changes associated with LRs? What other molecular and immune mechanisms might be linked to LRs? Ultimately, finding answers to these questions is essential for enhancing the quality of life for leprosy patients and preventing complications arising from these immune events.

## References

[bib1] Abbas A, Lichtman A, Pillai S. Cellular and molecular immunology [Internet]. Philadelphia, United States: Elsevier/Saunders. 2015, [cited 2017 Jan 14]. Available from: https://www.ncbi.nlm.nih.gov/nlmcatalog/101630458.

[bib2] Alizadeh A, Dyck SM, Karimi-Abdolrezaee S. Myelin damage and repair in pathologic CNS: challenges and prospects. Front Mol Neurosci. 2015;8:35. 26283909 10.3389/fnmol.2015.00035 PMC4515562

[bib3] Antunes DE, Ferreira GP, Nicchio MVC et al. Number of leprosy reactions during treatment: clinical correlations and laboratory diagnosis. Rev Soc Bras Med Trop. 2016;49:741–5. 28001221 10.1590/0037-8682-0440-2015

[bib4] Antunes DE, Goulart IMB, Lima MIS et al. Differential expression of IFN-γ, IL-10, TLR1, and TLR2 and their potential effects on downgrading leprosy reaction and erythema nodosum leprosum. J Immunol Res. 2019;2019:1. 10.1155/2019/3405103 PMC6875386 31781675

[bib5] Antunes DE, Santos DF, Lima MIS et al. Clinical, epidemiological, and laboratory prognostic factors in patients with leprosy reactions: A 10-year retrospective cohort study. Front Med. 2022;9:841030. 10.3389/fmed.2022.841030 PMC9358030 35957854

[bib6] Bahia El Idrissi N, Hakobyan S, Ramaglia V et al. Complement activation in leprosy: A retrospective study shows elevated circulating terminal complement complex in reactional leprosy. Clin Exp Immunol. 2016;184:338–46. 26749503 10.1111/cei.12767 PMC4872382

[bib7] Bahia El Idrissi N, Iyer AM, Ramaglia V et al. In Situ complement activation and T-cell immunity in leprosy spectrum: An immunohistological study on leprosy lesional skin. PLoS One. 2017;12:e0177815. 28505186 10.1371/journal.pone.0177815 PMC5432188

[bib8] Cardona-Castro N, Escobar-Builes MV, Serrano-Coll H et al. Mycobacterium lepromatosis as Cause of Leprosy, Colombia. Emerg Infect Dis. 2022;28:1067–8. 35450566 10.3201/eid2805.212015 PMC9045448

[bib9] Cruz VA, de Albuquerque CP, Guimarães MFB de R et al. New insights at the interface between leprosy and immune-mediated rheumatic diseases. Front Med. 2023;10:1239775. 10.3389/fmed.2023.1239775 PMC10564075 37822467

[bib10] de Barros B, Pierce R, Sprenger C et al. COVID-19 vaccination and leprosy-A UK hospital-based retrospective cohort study. PLoS Negl Trop Dis. 2023;17:e0011493. 37540711 10.1371/journal.pntd.0011493 PMC10431605

[bib11] Della Corte CM, Morgillo F. Early use of steroids affects immune cells and impairs immunotherapy efficacy. ESMO Open. 2019;4:e000477. 30964127 10.1136/esmoopen-2018-000477 PMC6435247

[bib12] de Oliveira AL, Amadeu TP, de França Gomes AC et al. Role of CD8(+) T cells in triggering reversal reaction in HIV/leprosy patients. Immunology. 2013;140:47–60. 23566249 10.1111/imm.12108 PMC3809705

[bib13] Deschênes J . Ocular Manifestations of Leprosy [Internet]. 2023. Available from: https://emedicine.medscape.com/article/1213853-overview?form=fpf.

[bib14] Dewi DAR, Djatmiko CBP, Rachmawati I et al. Immunopathogenesis of Type 1 and Type 2 Leprosy Reaction: An Update Review. Cureus. 2023;15:e49155. 38130570 10.7759/cureus.49155 PMC10733783

[bib15] Dupnik KM, Bair TB, Maia AO et al. Transcriptional changes that characterize the immune reactions of leprosy. J Infect Dis. 2015;211:1658–76. 25398459 10.1093/infdis/jiu612 PMC4425823

[bib16] Fonseca AB de L, Simon M do V, Cazzaniga RA et al. The influence of innate and adaptative immune responses on the differential clinical outcomes of leprosy. Infect Dis Poverty. 2017;6:5. [cited 2017 Jun 2]. Available from: http://www.ncbi.nlm.nih.gov/pmc/articles/PMC5292790/. 28162092 10.1186/s40249-016-0229-3 PMC5292790

[bib17] Frade MAC, Coltro PS, Filho FB et al. Lucio's phenomenon: A systematic literature review of definition, clinical features, histopathogenesis and management. IJDVL. 2022;88:464–77. 10.25259/IJDVL_909_19 34672479

[bib18] Froes LAR, Sotto MN, Trindade MAB. Leprosy: clinical and immunopathological characteristics. An Bras Dermatol. 2022;97:338–47. 35379512 10.1016/j.abd.2021.08.006 PMC9133310

[bib19] Galeano J, Contreras A, Pabón L et al. Case Report: Necrotizing Erythema Nodosum as a Manifestation of Lepromatous Leprosy Relapse 50 Years after the Initial Infection. Am J Trop Med Hyg. 2023;109:53–56. 37253443 10.4269/ajtmh.22-0701 PMC10323994

[bib20] Gautam S, Sharma D, Goel A et al. Insights into Mycobacterium leprae Proteomics and Biomarkers-An Overview. Proteomes. 2021;9:7. 33573064 10.3390/proteomes9010007 PMC7931084

[bib21] Gomes de Castro KK, Lopes da Silva PH, Nahar Dos Santos L et al. Downmodulation of Regulatory T Cells Producing TGF-β Participates in Pathogenesis of Leprosy Reactions. Front Med. 2022;9:865330. 10.3389/fmed.2022.865330 PMC9341400 35924037

[bib22] Gómez CH, Castro NMC. Reacciones leprosas. CES Med. 2016;30:200–9.

[bib23] Goulart IMB, Santana MA de O, Costa WVT d et al. Type 2 leprosy reaction presenting as a monoarthritis post multidrug therapy. IDCases. 2022;27:e01386. 35036324 10.1016/j.idcr.2022.e01386 PMC8749206

[bib24] Hafsi W, Badri T. Erythema Nodosum. In: StatPearls [Internet]. Treasure Island (FL): StatPearls Publishing; 2023; [cited 2023 Sep 13]. Available from: http://www.ncbi.nlm.nih.gov/books/NBK470369/.

[bib25] Jurado F, Rodriguez O, Novales J et al. Lucio's leprosy: a clinical and therapeutic challenge. Clin Dermatol. 2015;33:66–78. 25432812 10.1016/j.clindermatol.2014.07.004

[bib26] Kim BS, Kim JY, Lee JG et al. Immune modulatory effect of thalidomide on T cells. Transplant Proc. 2015;47:787–90. 25891732 10.1016/j.transproceed.2014.12.038

[bib27] Leon KE, Salinas JL, McDonald RW et al. Complex Type 2 Reactions in Three Patients with Hansen's Disease from a Southern United States Clinic. Am J Trop Med Hyg. 2015;93:1082–6. 26304919 10.4269/ajtmh.15-0052 PMC4703255

[bib28] Luo Y, Kiriya M, Tanigawa K et al. Host-Related Laboratory Parameters for Leprosy Reactions. Front Med. 2021;8:694376. 10.3389/fmed.2021.694376 PMC8568883 34746168

[bib29] Misra DP, Parida JR, Chowdhury AC et al. Lepra reaction with lucio phenomenon mimicking cutaneous vasculitis. Case Rep Immunol. 2014;2014:641989. 10.1155/2014/641989 PMC4280809 25580317

[bib30] Mitra D . Rare Atypical Presentations in Type 2 Lepra Reaction: A Case Series. Open Forum Infect Dis. 2017;4:S672. 10.1111/ijd.12292 24134145

[bib31] Mitra D . A Randomized Controlled Trial of Prednisolone vs. Interleukin 17 A Inhibitor Secuinumab in the Management of Type 1 Lepra Reaction in Leprosy Patients. 2018. Available from: https://www.ncbi.nlm.nih.gov/pmc/articles/PMC6254982/.

[bib32] Naafs B, van Hees CLM. Leprosy type 1 reaction (formerly reversal reaction). Clin Dermatol. 2016;34:37–50. 26773622 10.1016/j.clindermatol.2015.10.006

[bib33] Negera E, Walker SL, Girma S et al. Clinico-pathological features of erythema nodosum leprosum: A case-control study at ALERT hospital, Ethiopia. PLoS Negl Trop Dis. 2017;11:e0006011. 29028793 10.1371/journal.pntd.0006011 PMC5656324

[bib34] Nery JA da C, Bernardes Filho F, Quintanilha J et al. Understanding the type 1 reactional state for early diagnosis and treatment: A way to avoid disability in leprosy. An Bras Dermatol. 2013;88:787–92. 24173185 10.1590/abd1806-4841.20132004 PMC3798356

[bib35] Nickerson DS, Nickerson DE. A review of therapeutic nerve decompression for neuropathy in Hansen's disease with research suggestions. J Reconstr Microsurg. 2010;26:277–84. 20143300 10.1055/s-0030-1248237

[bib36] Nunzi E, Massone C, Portaels F. Leprosy and Buruly ulcer-A pracical guide. Second edition. Germany: Springer, 2020.

[bib37] Peixoto AB, Portela PS, Leal FRP de C et al. Rodrigues NC dos S. Lucio's phenomenon. Case study of an exceptional response to treatment exclusively with multibacillary multidrug therapy. An Bras Dermatol. 2013;88:93–96. 24346890 10.1590/abd1806-4841.20132398 PMC3876018

[bib38] Pinheiro JV, Pontes MA de A, Medeiros Neto JU d et al. Lucius phenomenon: the importance of a primary dermatological care. An Bras Dermatol. 2022;97:54–57. 34810028 10.1016/j.abd.2020.08.033 PMC8799846

[bib39] Pitta IJR, Hacker MA, Vital RT et al. Leprosy Reactions and Neuropathic Pain in Pure Neural Leprosy in a Reference Center in Rio de Janeiro—Brazil. Front Med. 2022;9:865485. 10.3389/fmed.2022.865485 PMC8992651 35402428

[bib40] Polycarpou A, Walker SL, Lockwood DNJ. A Systematic Review of Immunological Studies of *Erythema Nodosum Leprosum*. Front Immunol. 2017;8:233. 28348555 10.3389/fimmu.2017.00233 PMC5346883

[bib41] Putinatti MS de MA, Lastória JC, Padovani CR. Prevention of repeated episodes of type 2 reaction of leprosy with the use of thalidomide 100 mg/day. An Bras Dermatol. 2014;89:266–72. 24770503 10.1590/abd1806-4841.20142037 PMC4008057

[bib42] Putri AI, de Sabbata K, Agusni RI et al. Understanding leprosy reactions and the impact on the lives of people affected: an exploration in two leprosy endemic countries. PLoS Negl Trop Dis. 2022;16:e0010476. 35696438 10.1371/journal.pntd.0010476 PMC9191760

[bib43] Ranugha P, Chandrashekar L, Kumari R et al. Is it Lucio Phenomenon or Necrotic Erythema Nodosum Leprosum?. Indian J Dermatol. 2013;58:160. 10.4103/0019-5154.108087 PMC3657244 23716834

[bib44] Rathod SP, Jagati A, Chowdhary P. Disabilities in leprosy: an open, retrospective analyses of institutional records. An Bras Dermatol. 2020;95:52–56. 31952993 10.1016/j.abd.2019.07.001 PMC7058852

[bib45] Rocha RH, Emerich PS, Diniz LM et al. Amaral ACV do. Lucio's phenomenon: exuberant case report and review of Brazilian cases. An Bras Dermatol. 2016;91:60–63. 28300896 10.1590/abd1806-4841.20164370 PMC5324995

[bib46] Safa G, Darrieux L, Coic A et al. Type 1 leprosy reversal reaction treated with topical tacrolimus along with systemic corticosteroids. Indian J Med Sci. 2009;63:359–62. 19770527

[bib47] Schmitz V, Tavares IF, Pignataro P et al. Neutrophils in Leprosy. Front Immunol. 2019;10:495. 30949168 10.3389/fimmu.2019.00495 PMC6436181

[bib48] Schomberg D, Ahmed M, Miranpuri G et al. Neuropathic pain: role of inflammation, immune response, and ion channel activity in central injury mechanisms. Ann Neurosci. 2012;19:125–32. 25205985 10.5214/ans.0972.7531.190309 PMC4117080

[bib49] Scollard DM . The biology of nerve injury in leprosy. LEPROSY. 2008;79:242–53. 19009974

[bib50] Sehgal VN . Lucio's phenomenon/erythema necroticans. Int J Dermatology. 2005;44:602–5. 10.1111/j.1365-4632.2005.02567.x 15985035

[bib51] Serrano-Coll H, Salazar-Peláez L, Acevedo-Saenz L et al. Mycobacterium leprae-induced nerve damage: direct and indirect mechanisms. Pathog Dis. 2018;76. 10.1093/femspd/fty062. 30052986

[bib52] Sharma P, Kumar A, Tuknayat A et al. Phenomenon: A Rare Presentation of Hansen's Disease. J Clin Aesthetic Dermatol. 2019;12:35–8. PMC7002045 32038763

[bib53] Singh SK, Sharma T, Rai T et al. Type 2 lepra reaction in an immunocompromised patient precipitated by filariasis. Indian J Sex Transm Dis AIDS. 2014;35:40–2. 24958985 10.4103/2589-0557.132418 PMC4066596

[bib54] Stefani MM, Guerra JG, Sousa ALM et al. Potential plasma markers of Type 1 and Type 2 leprosy reactions: a preliminary report. BMC Infect Dis. 2009;9:75. 19473542 10.1186/1471-2334-9-75 PMC2696458

[bib55] Tajalli M, Wambier CG. Lucio's Phenomenon. N Engl J Med. 2021;384:1646. 33913641 10.1056/NEJMicm2025081

[bib56] Talhari S, Garrido N, Oliveira G et al. Hanseníase. 4th ed. Manaus, Brazil, 2006, 216p.

[bib57] Thangaraju P, Venkatesan S, Gurunthalingam M et al. Rationale use of Thalidomide in erythema nodosum leprosum—A non-systematic critical analysis of published case reports. Rev Soc Bras Med Trop. 2020;53:e20190454. 32935774 10.1590/0037-8682-0454-2019 PMC7491565

[bib58] Upputuri B, Pallapati MS, Tarwater P et al. Thalidomide in the treatment of erythema nodosum leprosum (ENL) in an outpatient setting: a five-year retrospective analysis from a leprosy referral centre in India. PLoS Negl Trop Dis. 2020;14:e0008678. 33035210 10.1371/journal.pntd.0008678 PMC7577491

[bib59] Vashisht D, Neema S, Tripathy DM et al. Bullous Erythema Nodosum Leprosum Through the Dermoscope. Dermatol Pract Concept. 2022;12:e2022027. 35223171 10.5826/dpc.1201a27 PMC8825209

[bib60] Velarde-Félix JS, Alvarado-Villa G, Vera-Cabrera L. “Lucio's Phenomenon” Associated with Mycobacterium lepromatosis. Am J Trop Med Hyg. 2016;94:483–4. 26936990 10.4269/ajtmh.15-0439 PMC4775876

[bib61] Wu J, Boggild AK. Clinical Pearls: Leprosy Reactions. J Cutan Med Surg. 2016;20:484–5. 27060010 10.1177/1203475416644832

[bib62] Ya SNC, Muhamad R, Zakaria R et al. Lucio Phenomenon: Sequelae of Neglected Leprosy. Korean J Fam Med. 2021;42:245–9. 31968408 10.4082/kjfm.19.0068 PMC8164933

